# miR-199a-5p confers tumor-suppressive role in triple-negative breast cancer

**DOI:** 10.1186/s12885-016-2916-7

**Published:** 2016-11-14

**Authors:** Jiawei Chen, Vivian Y. Shin, Man T. Siu, John C. W. Ho, Isabella Cheuk, Ava Kwong

**Affiliations:** 1 Breast Surgery Division, Department of Surgery, The University of Hong Kong, Hong Kong, SAR China; 2 Hong Kong Hereditary Breast Cancer Family Registry, Queen Mary Hospital, Room K1401, Pokfulam Road, Pok Fu Lam, Hong Kong; 3 Department of Surgery, Hong Kong Sanatorium and Hospital, Hong Kong, SAR China

**Keywords:** miR-199a-5p, Triple negative breast cancer (TNBC), Tumor-suppressor

## Abstract

**Background:**

Triple-negative breast cancer (TNBC) remains a poor prognostic factor for breast cancer since no effective targeted therapy is readily available. Our previous studies confirmed miR-199a-5p is a TNBC-specific circulating biomarker, however, its functional roles in breast cancer is largely unknown. Thus, we investigated the functional implication of miR-199a-5p in TNBC and its potential underlying mechanisms.

**Methods:**

MTT assay was performed to investigate the cell proliferation after transient transfection of miR-199a-5p in MDA-MB-231 cell line, followed by cell cycle analysis. Transwell invasion assay and wound healing assay were used to study the invasion and migration ability respectively. To further investigate the stemness-related characteristics of miR-199a-5p in breast cancer cells, single-cell clonogenic assay and aldehyde dehydrogenase (ALDH) assay were performed. 32 normal and 100 breast cancer patients’ plasma were recruited to identify the potential circulating markers by qPCR.

**Results:**

Cell proliferation assay revealed significant inhibition after miR-199a-5p ectopic expression (*p* < 0.0001), as a result of decreased S phase (*p* = 0.0284), increased G0/G1 phase (*p* = 0.0260) and apoptosis (*p* = 0.0374). Invasiveness (*p* = 0.0005) and wound healing ability were also decreased upon miR-199a-5p overexpression. It significantly altered EMT-related genes expression, namely CDH1, ZEB1 and TWIST. Single-cell clonogenic assay showed decreased colonies in miR-199a-5p (*p* = 0.0182). Significant downregulation (*p* = 0.0088) and inhibited activity (*p* = 0.0390) of ALDH was observed in miR-199a-5p. ALDH1A3, which is the dominant isoform of ALDH, is significantly upregulated in breast cancer plasma especially in TNBC (*p* = 0.0248). PIK3CD was identified as a potential downstream target of miR-199a-5p.

**Conclusions:**

Taken together, we unraveled, for the first time, the tumor-suppressive role of miR-199a-5p in TNBC, which attributed to EMT and cancer stemness properties, providing a novel therapeutic options towards this aggressive disease.

## Background

Breast cancer is one of the most frequent cancers in the world with approximately 1.67 million new cases diagnosed in 2012 [Globocan 2012, http://globocan.iarc.fr/Pages/fact_sheets_cancer.aspx]. It is the second most common cause of cancer-related mortality among women, accounting for over 500,000 deaths every year [1]. Like other cancers, breast cancer is a heterogeneous tumors originating from mammary epithelial cells with a high degree of diversity between and within tumors as well as among cancer-bearing individuals [2]. The classification of subtypes is widely applied in clinical settings based on the human epidermal growth factor receptor 2 (HER2) expression, hormonal receptors including estrogen receptor (ER), progesterone receptor (PR). They are classified into different subtypes: luminal A (ER + and/or PR+, Ki67 low and HER2-), luminal B (ER + and/or PR+, Ki67 high and/or HER2+), HER2-positive (ER-, PR- and HER2+) and triple-negative (ER-, PR-, HER2-). TNBC accounts for only 15–20% of all breast cancer cases while exhibiting the most aggressive biological phenotype and the metastasis rate is 35% after 6-year follow-up [3]. Also, TNBC had an increased likelihood of distant recurrence and mortality within 5 years of diagnosis when compared to the non-TNBC patients. Due to the absence of ER, PR and HER2, TNBC is unresponsive to treatment targeting these receptors such as Tamoxifen (an antagonist to the ER), or Herceptin (monoclonal antibody against HER2 receptor).

MicroRNAs (miRNAs) are classified as small non-coding regulatory RNAs (18–24 nucleotides in length) that are emerged as central posttranscriptional repressors of gene expression. MiRNAs regulate numerous biological processes such as cell growth, development and differentiation by determining the “on-and-off” of the genes expression [4]. Recent studies have demonstrated a fundamental role of miRNAs in the development of various diseases involved different systems such as neurology, cardiovascular, endocrine and developmental diseases [5]. Alterations in miRNA expression have been linked to the initiation and development of cancers [6]. In clinical setting, some breast cancer patients with similar disease characteristics show different clinical outcomes, indicating the needs for an affordable, reliable, less invasive approach to predict the diagnosis and prognosis of breast cancer. It has been demonstrated that miRNAs play a role in oncogenesis, metastasis, and resistance in cancer, they can be further classified as oncogenes or tumor-suppressor genes [7–9]. Some miRNAs, for example, miR-10b, miR-21 and miR-155 confer their oncogenic role by deactivating tumor-suppressor genes while activating oncogenic transcription factors in breast cancer [7, 10]. A majority of miRNAs exhibit downregulation in breast cancer, indicating their tumor-suppressive function, for example, miR125b, miR200, miR206 [11–14]. In this regard, miRNAs have been indicated as potential biomarkers as they can be readily detected in breast cancer tissues [15] and also stably expressed in human body fluids such as plasma, serum and saliva [16].

Accumulating evidence demonstrated that miRNAs show distinct expression signatures across different cancer subtypes, and have either oncogenic or tumor-suppressive roles [17, 18]. Our previous study discovered that miR-199a-5p showed low expression in TNBC patients’ circulation when compared with other subtypes of breast cancers, also the expression level was linked with disease stage, and suggested to be a potential biomarker for TNBC [19]. In 2003, miR-199a-5p was first described to be originated from two genetic loci. One was chromosome 19 for miR-199a-1 and the other one was chromosome 1 for miR-199a-2. It is reported that miR-199a-5p inhibited cell proliferation and induced cell death [20, 21]. Based on these findings, we sought to investigate the functional implications of miR-199a-5p in breast cancer both in vivo and in vitro.

## Methods

### Patients

We recruited 100 breast cancer patients (50 TNBC and 50 non-TNBC) from different hospitals namely Queen Mary Hospital, the Tung Wah Hospital, the Hong Kong Sanatorium and Hospital and the Hong Kong Hereditary Breast Cancer Family Registry. Written consent forms were obtained from all participating patients. The clinical characteristics of patients were listed as in Table 1. We also recruited 32 healthy individuals without personal history of cancers including breast cancer from the Queen Mary Hospital and the Tung Wah Hospital as normal control cases. The approval of the study was obtained through Institutional Review Board of the University of Hong Kong and Hong Kong Sanatorium and Hospital (No. UW 14–441).

**Table 1:** Clinical characteristics of TNBC and non-TNBC breast cancer patients

	Non-TNBC (*n* = 50)	TNBC (*n* = 50)	*P*-value
Age (mean, s.d.)	50.7 (12.5)	56.4 (14.1)	0.0346
Histological type			
Grade 1, 2	28	12	0.0067
Grade 3	15	25	
NA	7	13	
Metastasis			0.0309
No	49	42	
Yes	1	8	
Stage			n.s.
0, I, II	44	40	
III, IV	5	10	
NA	1	0	
Bilateral			n.s.
No	48	47	
Yes	2	3	
Histological type			n.s.
DCIS	5	1	
IDC	42	42	
Mixed (IDC + ILC)	1	2	
Others	2	5	

*DCIS* ductal carcinoma in situ; *IDC* invasive ductal carcinoma; *ILC* invasive lobular carcinoma; *NA* not applicable; *n.s*. not significant

### Cell culture and transfection

A metastatic human breast cancer cell line MDA-MB-231 (HTB-26) was purchased from the American Type Culture Collection (Manassas, VA) and cultured in 37°C incubator with RPMI-1640 medium (Invitrogen, NY, USA) supplemented with 10% fetal bovine serum. Cell line was used in less than 6 months of continuous passage after acquisition and authenticated by the cell bank source using short tandem repeat profiling. Cells were transfected with Allstar Negative Control siRNA and hsa-miRNA-199a-5p mimic (Qiagen, CA, USA) for 72 h and collected for further studies. To develop the stable cell line, hsa-miR-199a precursor with 200–250 bp of flanking sequences were PCR amplified from human genomic DNA (forward primer sequence: GACTAAGCTTAGCAGAAGCCACGATCCCAAAC; reverse primer sequence: GACTGGATCC GGATGGCAGACTGATAGGGC), and cloned into the pmR-ZsGreen1 Vector (Clontech, CA, USA). The insert was verified by Sanger Sequencing, and the expression plasmid was transfected into MDA-MB-231 cells using Lipofectamine 3000 (Life Technologies, CA, USA) for miRNA expression. Stable cell clones were selected with 0.5 mg/ml Geneticin (Life Technologies, CA, USA), and the expression of miR-199a-5p was confirmed by qPCR.

### Cell proliferation assay

Stable transfected cells (3×10^3^) were seeded in a 96-well microtiter plate with triplicate wells. After incubation for 3 days, MTT assay was used to measure cell viability from day1 to day5, intracellular purple formazan was solubilized in DMSO followed by the colorimetric product quantified at absorbance 570 nm.

### Migration assay

Cell migration ability was investigated using scratch wound-healing assay. After cells reached 90% confluent in a 6-well plate, a sterile pipette tip was used to scratch on cell monolayer. The wound area was monitored under microscopy (Olympus CKX41, MA, USA) and the images were acquired for each sample at 0, 3, 9 and 12 h by DP controller 3.31.292 (Olympus, MA, USA).

### Cell invasion assay

Cell invasion assays were performed using the BD BioCoat™ Matrigel™ Invasion Chamber (BD Biosciences, MA, USA) according to the manufacturer’s instructions. Briefly, MDA-MB-231 cells were transfected with miR-199a-5p, 3 days later, 2.5×10^4^ cells resuspended in serum-free RPMI 1640 medium were placed in the upper chambers, and chemoattractant (RPMI 1640 medium supplemented with 10% fetal bovine serum) was placed in the lower chamber, and cells were allowed to invade for 24 h at 37°C. Invaded cells were fixed with 100% methanol and stained with crystal violet. Invaded cells were counted at magnification x400 from 10 random fields. All experiments were performed in triplicates.

### Single-cell clonogenic assay

Single-cell clonogenic assay was performed to investigate the self-renewal ability in vitro. Briefly, we seeded each well with one cell in the 96 well plates and cultured in complete medium. The number of cell colonies (greater than 50 cells) were counted after 7 days.

### RNA extraction and quantitative RT-PCR (qRT-PCR)

Purification of total RNA was performed by using the miRNeasy Serum/Plasma Kit (Qiagen, Hilden, Germany) according to the manufacturer’s protocol. Briefly, 1 ml QIAzol Lysis Reagent was added to 200 μl plasma. Then 200 μl chloroform was added for separation followed by adding 100% ethanol to the aqueous phase. After being mixed thoroughly, sample was transferred into an RNeasy MinElute spin column for centrifugation. The concentrations of eluted RNA samples were analyzed by NanoDrop 1000 (Thermo Scientific, DE, USA). Reverse transcription was done by Superscript® IV Reverse Transcriptase (Invitrogen, CA, USA) according to manufacturer’s standard protocol. Light Cycler 480 SYBR Green I Master (Roche, Mannheim, Germany) was used to perform real-time qPCR in Roche LC480 machine.

### Immunofluorescence

Cells were seeded on the cover slips and incubated for 24 h. Cells were washed with PBS and fixed in 2% paraformaldehyde for 20 min in room temperature. Then cells were permeabilized by 0.2% triton/PBS for 10 min in room temperature. After washing with PBS, 3% BSA/PBS was added to cells to block the non-specific binding sites for 45 min and incubated with anti-Twist (Abcam, Cambridge, UK) or anti-E-cadherin (Cell Signaling, MA, USA) antibody for 60 min, followed by incubation with secondary donkey anti-rabbit antibody (Abcam, Cambridge, UK) for 50 min. After washing, 4′, 6-diamidino-2-phenylindole (DAPI) was used to stain the nucleus and were observed under immunofluorescence microscopy (Nikon, Eclipse 80i, Tokyo, Japan).

### Aldehyde dehydrogenase (ALDH) activity

ALDH activity was measured by using the ALDEFLUOR™ Assay System (StemCell Technologies, WA, USA) according to the manufacturer’s recommendations. Single cells were resuspended in ALDEFLUOR™ Assay Buffer and incubated with the activated ALDEFLUOR™ Reagent, biodipy-aminoacetaldehyde (BAAA). For negative control, an equal amount aliquot of cells was also incubated with the activated ALDEFLUOR™ Reagent and the specific ALDH inhibitor, diethylaminobenzaldehyde (DEAB). After 40 min, cells were washed with PBS and resuspended in ALDEFLUOR™ Assay Buffer in 4°C. Cells were analyzed using a dual laser BD FACS Calibur (BD Biosciences, MA, USA).

### CD24-/CD44+ cell population analysis

Combinations of fluorochrome-conjugated monoclonal antibodies against human CD44 (FITC, 555478, BD Pharmingen™, CA, USA) and CD24 (PE, 555428, BD Pharmingen™, CA, USA) or their respective controls were added to the cell suspension at recommended concentrations as suggested by the manufacturer instruction and incubated at 4°C in dark for 45 min. After incubation, cells were fixed with 2% paraformaldehyde and analyzed using BD FACS Calibur (BD Biosciences, MA, USA).

### Cell cycle analysis

3 ml ice-cold 70% ethanol was added to fix cells at −20°C overnight. After fixation, cell pellets were collected by centrifugation. After washed three times by PBS, cells were stained with 20 μg/ml propidium iodide and 0.2 mg/ml RNase A for 30 min. BD FACSCalibur (BD Biosciences, MA, USA) was used to perform flow cytometry and Cell Quest software for data analysis. The experiments were performed in triplicates.

### Apoptosis assay

Cellular apoptosis was detected by FITC Annexin V Apoptosis Detection Kit (BD Pharmingen™, CA, USA) according to manufacturer’s standard protocol. Briefly, cells were suspended in 1X binding buffer at concentration of 1×10^6^ cells/ml. 5 μl FITC Annexin V together with 5 μl PI were added to 100 μl of resuspended cells and gently vortexed for 15 min at room temperature in dark. After incubation, 400 μl 1X binding buffer was added then analyzed by BD FACSCalibur (BD Biosciences, MA, USA).

### Immunohistochemistry

TWIST and CDH1 expressions were detected in formalin-fixed, paraffin-embedded tissue sections according to the manufacturer’s instructions. Briefly, tissue sections were deparaffinized and rehydrated, followed by an epitope retrieval step incubated in a citrate buffer solution at 90–95°C for 10 min prior to 3% hydrogen peroxide. Slides were then blocked with 5% blocking solution for 1 h at room temperature and incubated with anti-CDH1, (3195S, Cell Signaling Technologies, MA, USA) and anti-TWIST (ab50581, Abcam, Cambridge, UK) antibody overnight at 4°C. Visualization was performed by SignalStain® Boost Detection Reagent (Cell Signaling Technologies, MA, USA) for 30 min. Slide images were obtained using an Eclipse E600 microscope (Nikon, NY, USA).

### Dual-luciferase assay

Potential target genes of hsa-miR-199a-5p were predicted in silico using online algorithms, including: miRDB (http://mirdb.org/miRDB/index.html) and miRmap (http://mirmap.ezlab.org/). 3′-untranslated regions (UTR) of human TGF-β2 and PIK3CD, which contain the putative miRNA target sites, were PCR amplified (TGF-β2-3′UTR forward primer sequence: GACTGCTAGCTAAAATTCTTGGAAAAGTGGCA, reverse primer sequence: GACTGTCGACGGTCATATAATAACTCACTTGG; PIK3CD-3′UTR forward primer sequence: GACTGCTAGCAAGACAACAGGCAGTAGTGGCT, reverse primer sequence: GACTGTCGACCAGCGTAGATTCTCCTTT) from MDA-MB-231 cDNA and cloned into the pmirGLO Dual-Luciferase miRNA Target Expression Vector (Promega, MI, USA) between NheI and SalI sites. TGF-β2-, PIK3CD- and empty pmirGLO reporter constructs were co-transfected with miR-199a-5p mimics or mimic negative control (Qiagen, CA, USA), to a final concentration of 10 nM, into MDA-MB-231 cells using Lipofectamine 3000 (Invitrogen, CA, USA) for 48 h. Relative luciferase activities were determined using Dual-Glo Luciferase Assay System (Promega, MI, USA).

### In vivo xenograft study

Briefly, 1×10^6^ miR-199a-5p overexpressing cells and control cells were injected into the nude mice fat pad. Tumor sizes were calculated using formula 1/2(length × width^2^) every 7 days. Tumors were harvested for measurement after the mice were sacrificed at week 5.

### Statistical analysis

The differences between groups were estimated by Student’s *t*-test, non-parametric Mann-Whitney *U* test, Chi-square test as appropriate. *P* < 0.05 was considered as statistically significant. All statistical analyses were performed using GraphPad Prism 6.0 (GraphPad Software Inc., San Diego, CA, USA).

## Results

### miR-199a-5p inhibited cell proliferation by inducing G0/G1 phase arrest and apoptosis in breast cancer cells

**Fig. 1:**
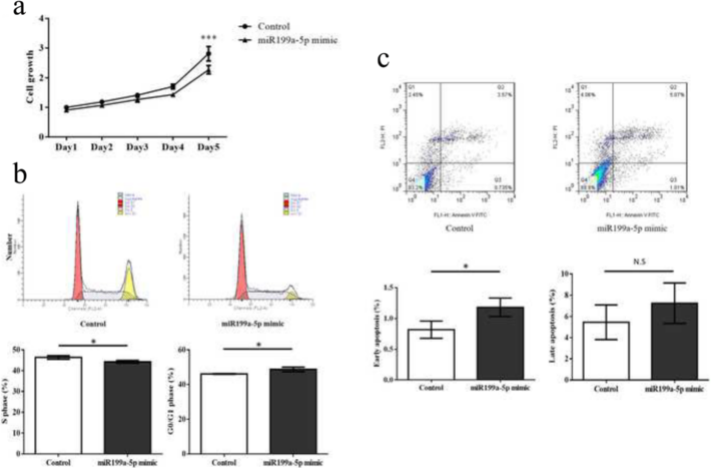
miR-199a-5p inhibited cell proliferation. **a** MTT assay showed a significant inhibition on cell proliferation in miR-199a-5p overexpression group. **b** Cell cycle analysis showed a significant reduction in S phase (*p* = 0.0284) and increased G0/G1phase arrest (*p* = 0.0260). **c** Annexin V analysis showed that miR-199a-5p increased early apoptosis (*p* = 0.0374) (ns = not significant, *P ≤ 0.05, ****P ≤ 0.0001). All experiments were repeated in triplicates

To gain insight into the functional role of miR-199a-5p in breast cancer, MDA-MB-231 cells were transfected with miR-199a-5p mimic. Results showed that miR-199a-5p significant reduced cell proliferation by MTT assay (Fig. 1a). Cell cycle analysis showed that miR-199a-5p overexpression led to significant decrease in S-phase (*p* = 0.0284) and caused G0/G1 phase arrest (*p* = 0.0260) (Fig. 1b). Ectopic expression of miR-199a-5p showed significantly increased in early apoptosis (*p* = 0.0374) while no significant difference observed in late apoptotic cells (Fig. 1c).

### miR-199a-5p significantly inhibited cell migration and invasion ability in breast cancer cells through epithelial-mesenchymal transition (EMT) process

**Fig. 2:**
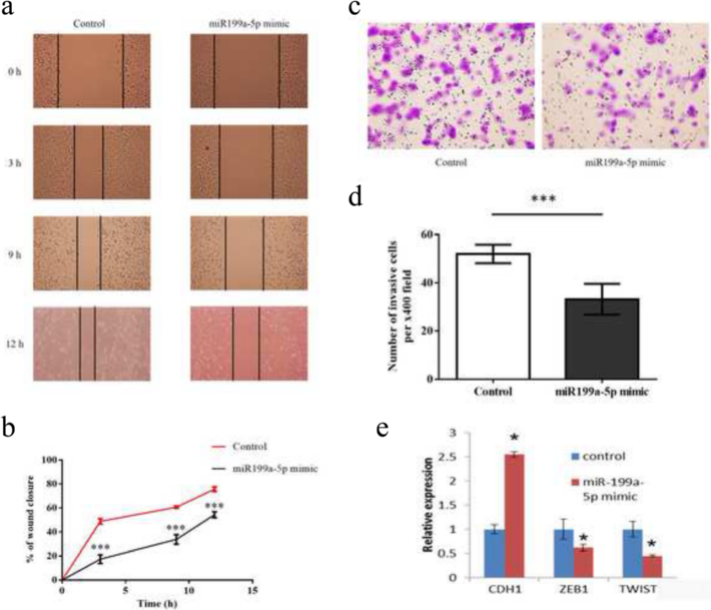
miR-199a-5p inhibited cell migration and invasion ability in breast cancer cells (**a**, **b**) In vitro wound healing process was mimicked by scratching cells surface. The rate of migration was measured by quantifying the total distance that the cells moved from the edge of the scratch toward the center of the scratch (marked by imaginary lines). Cells were observed under x200 microscope at different time points (0, 3, 9 and 12 h. **c**, **d** Transwell invasion assay showed miR-199a-5p significant decreased the number of invasive cells (52 vs 33 cells per x400 field in control and miR-199a-5p mimic group, *p* = 0.0005). **e** Ectopic expression of miR-199a-5p significantly upregulated CDH1 expression and downregulated ZEB1, TWIST expression. All experiments were repeated in triplicates

**Fig. 3:**
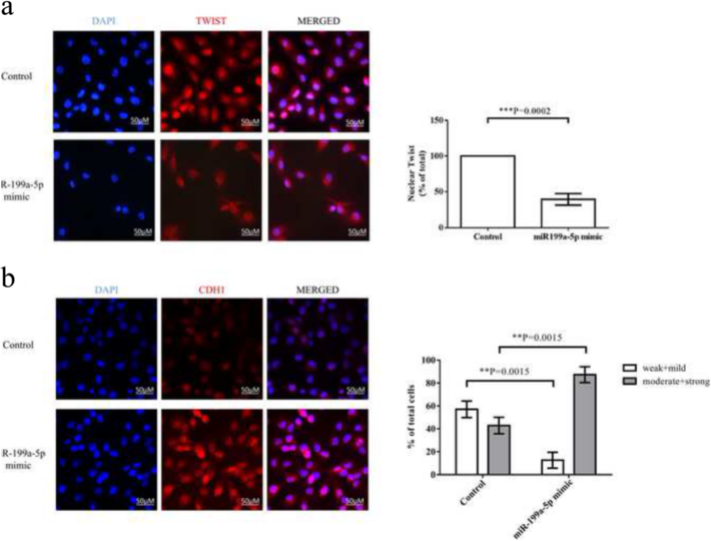
Immunofluorescent staining of TWIST (**a**) and CDH1 (**b**) in MDA-MB-231 and miR-199a-5p mimic. The result showed that miR-199a-5p induced obvious TWIST translocation from nucleus to cytoplasm (*p* = 0.0002) and more profound CDH1 expression compared to the control group (*p* = 0.0015). (*P ≤ 0.05, ***P ≤ 0.001). All experiments were repeated in triplicates

To study the effect of miR-199a-5p on EMT process, wound healing and invasion assays were assessed. In wound healing assay, the cell migration ability was significantly inhibited in miR-199a-5p overexpressing cells when compared with the control at different time points (3, 9, 12 h) (Fig. 2a & b). Invasion assay also demonstrated a profound decreased number of invasive cells in miR-199a-5p overexpressing cells (*p* = 0.0005) (Fig. 2c & d). Overexpression of miR-199a-5p altered the expression levels of EMT-related markers such as CDH1 (E-cadherin), ZEB1 and TWIST (Fig. 2e). Immunofluorescent staining result showed that miR-199a-5p induced obvious TWIST translocation from nucleus to cytoplasm (*p* = 0.0002) and profound CDH1 expression compared to the control group (*p* = 0.0015) (Fig. 3a, b). These results indicating that miR-199a-5p suppressed EMT process in breast cancer.

### miR-199a-5p inhibited stem-cell like properties in breast cancer cells

**Fig. 4:**
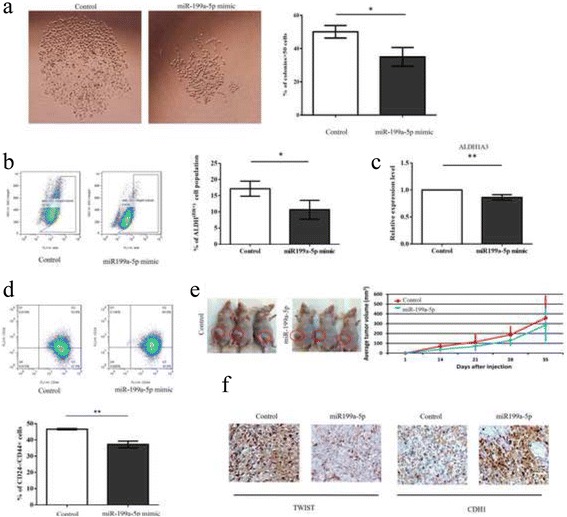
miR-199a-5p inhibited stem-cell like properties in breast cancer cells (**a**) Ectopic expression of miR-199a-5p reduced the single cell colony formation ability in MDA-MB-231 breast cancer cells (*p* = 0.0182). Single-cell clonogenic assay was used to determine single cell colony formation ability, cell clones reached more than 50 cells were considered as cell colonies. Representative micrographs (left panel) of colonies in single-cell clonogenic assay. **b** ALDH activity assay showed a significant reduced ALDH-positive cell population in miR-199a-5p mimic group than in the control group (17.2% vs 10.7%, *p* = 0.039). **c** ALDH1A3 expression was significantly suppressed after miR-199a-mimic treatment (*p* = 0.0088). **d** CD24-/CD44+ cell population showed significant decrease in miR-199a-5p overexpressing cells compared to its control group (*p* = 0.0015). **e** miR-199a-5p inhibited in vivo tumor growth. Left panel: three represented nude mice showed the reduction of tumor growth by overexpression of miR-199a-5p (control: left side, miR-199a-5p: right side). Right panel demonstrated the increase in volume of xenograft in the miR-199a-5p group. **f** Immunohistochemistry staining of mice xenograft from fat pad injection with MDA-MB-231 miR-199a-5p stable cells. (*P ≤ 0.05, **P ≤ 0.01). All experiments were repeated in triplicates

In order to explore the potential role of miR-199a-5p in stemness features, we examined the effect of miR-199a-5p in single-cell clonogenic assay. Ectopic expression of miR-199a-5p reduced the number of colonies (*p* = 0.0182) and their sizes, implicating the tumor suppressive role of miR-199a-5p on self-renewal ability (Fig. 4a). We further confirmed that ALDH activity significantly decreased in miR-199a-5p overexpressing cells (*p* = 0.039) when compared to control, indicating that miR-199a-5p played a role in stemness properties in breast cancer (Fig. 4b). Also, ALDH1A3 expression was lowered in miR-199a-5p mimic transfected cells (*p* = 0.0088) (Fig. 4c). We also confirmed that miR-199a-5p significantly decreased the CD24-/CD44+ cell population (*p* = 0.0015) (Fig. 4d), which is a well-reported subpopulation of breast cancer cells to have stem/progenitor cell properties. In line with the finding in vitro, miR-199a-5p overexpressing mice had smaller tumors than control mice (Fig. 4e). MiR-199a-5p downregulated TWIST and led to translocation from nucleus to cytoplasm, and induced CDH1 expression in xenograft tissues by immunostaining (Fig. 4f).

**Fig. 5:**
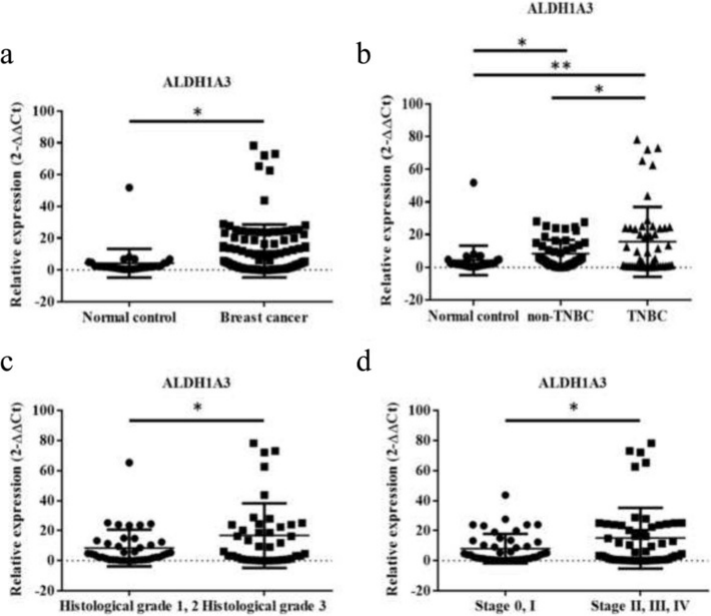
Expression of ALDH1A3 in 32 normal control and 100 breast cancer patients’ plasma. **a**, **b** Expression of ALDH1A3 significant elevated in breast cancer plasma (*p* < 0.0001), especially in TNBC. **c**, **d** ALDH1A3 significantly upregulated in breast cancer cases with higher histological grade (grade 3) (*p* = 0.0355) and more advanced disease stage (stage II, III, IV) (*p* = 0.0368). (*P ≤ 0.05, **P ≤ 0.01)

Moreover, ALDH1A3 significantly upregulated in the plasma of breast cancer patients, especially in TNBC when compared with normal control (Normal control vs breast cancer, *p* = 0.0135; non-TNBC vs TNBC, *p* = 0.0248) (Fig. 5a, b). Further stratification by clinical-pathological data showed that ALDH1A3 expression correlated with higher histological grade (*p* = 0.0355) and more advanced disease stage (*p* = 0.0368) (Fig. 5c, d). All these findings indicate that miR-199a-5p is related to stemness features in TNBC through regulation of ALDH1A3.

### miR-199a-5p influenced expression of TGF-β2 and PIK3CD in breast cancer

For identification of potential downstream targets of miR-199a-5p, several online prediction tools (miRDB, miRMAP) were used. All the predicted targets have prediction scores range from 50 to 100. These scores are assigned by the computational target prediction algorithm. Putative miR-199a-5p targets, PIK3CD and TGF-β2, were selected based on their high scores in all these two prediction tools (score: 94-99).

**Fig. 6:**
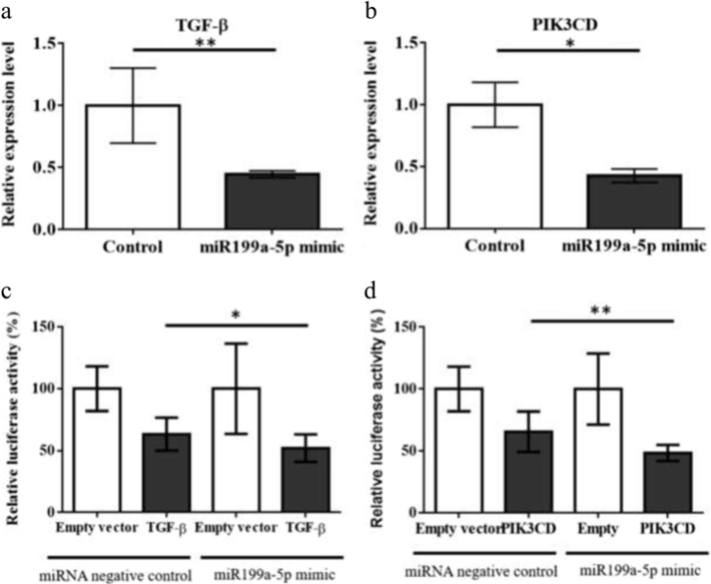
TGF-β2 and PIK3CD are potential downstream targets of miR-199a-5p. **a**, **b** Transient transfection of miR-199a-5p in MDA-MB-231 cell line significantly impeded TGF-β2 (*p* = 0.0317) and PIK3CD expression (*p* = 0.0079). **c**, **d** Luciferase reporter assay led to more profound reduction luciferase activity level of TGF-β2 (*p* = 0.0341) and PIK3CD (*p* = 0.0028) in miR-199a-5p mimic group than control group. (*P ≤ 0.05, **P ≤ 0.01). All experiments were repeated in triplicates

MiR-199a-5p mimic significantly downregulated TGF-β2 (*p* = 0.0317) and PIK3CD expression (*p* = 0.0079) in MDA-MB-231 breast cancer cell line (Fig. 6a, b). By dual luciferase reporter assay, mimic of miR-199a-5p decreased the relative luciferase activity in a greater extend in MDA-MB-231 transfected with reporter constructs containing the 3′-UTRs of PIK3CD and TGF-β2 compared to the empty construct controls (Fig. 6c, d) (*p* = 0.0341 in TGF-β2 group, *p* = 0.0028 in PIK3CD group), indicating TGF-β2 and PIK3CD are the direct downstream targets of miR-199a-5p.

**Fig. 7:**
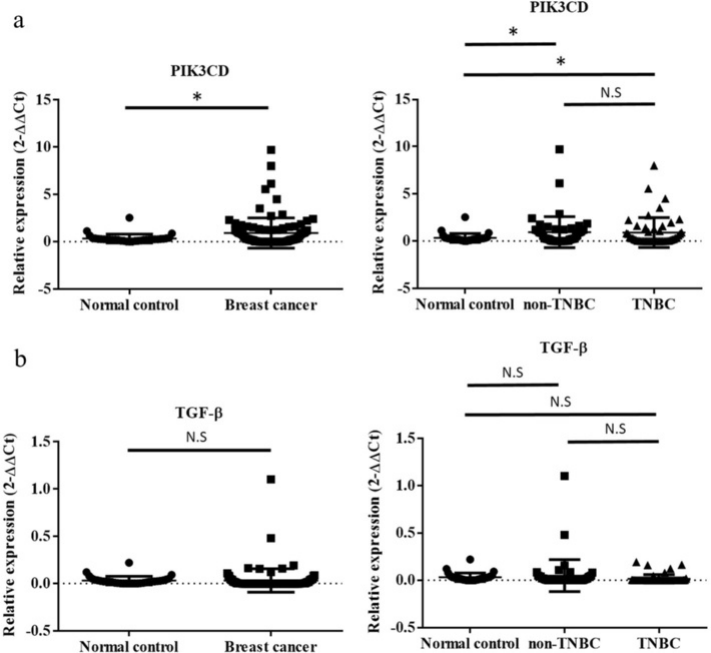
Expression of PIK3CD in 32 normal control and 100 breast cancer patients’ plasma. **a** PIK3CD showed upregulation in breast cancer circulation than normal control (*p* = 0.0453), but no obvious difference between non-TNBC and TNBC group. **b** TGF-β showed no difference between breast cancer circulation and normal control (ns = not significant, *P ≤ 0.05)

We further examined the gene expression in plasma of breast cancer patients. Results suggested that PIK3CD expression were higher in the circulation (*p* = 0.0453) (Fig. 7a) and TGF-β2 had no observable changes (Fig. 7b) in breast cancer patients when compared to normal control. However, neither genes exhibited significant different expression between non-TNBC and TNBC. The results indicated that PIK3CD is associated with breast cancer.

## Discussion

Our previous study demonstrated that miR-199a-5p expression was downregulated in TNBC when compared to other subtypes [19]. However, the functional roles and potential mechanism are still remained largely elusive. Our results also demonstrated that miR-199a-5p retarded proliferation, migration, invasion and stem cell-like characteristics in breast cancer. Recent studies revealed that miR-199a-5p exhibited downregulation and tumor-suppressive role in various types of cancers both in vitro and in vivo [22]. Our cell cycle and apoptosis assay results indicated that decreased S phase cells, G0/G1 phase arrest and enrichment of late apoptotic cells may be the potential mechanisms of the inhibitory role of miR-199a-5p. Accumulating evidence have established an important relationship between EMT and the acquisition of molecular and functional characteristics of cancer stem cells [23, 24]. The reduction of migration and invasion ability may be partly due to the altered EMT-related signaling, such as CDH2 by regulating transcriptional factor SNAI1 [25]. Similar observation was found in breast cancer cells, we discovered that miR-199a-5p greatly suppressed the migration and invasion ability. Some EMT-related genes such as CDH1, ZEB1 and TWIST were significantly altered by the introduction of miR-199a-5p in MDA-MB-231 cells, implicating the possibility of EMT process involvement. TWIST has been considered as a critical EMT inducer which allows the acquisition of mesenchymal phenotype that permit the invasion and metastasis from the primary tumor site [26, 27]. TWIST expression caused the inhibition of CDH1 and promoted the expression of fibroblastic markers such as fibronectin, smooth-muscle actin and vimentin [28, 29]. In gastric cancer, a high TWIST protein expression level was associated with a reduction in CDH1 protein and with the increased CDH2 which may enhance cell motility in several cancer types [29]. Consistent with the above findings, our result shed light into the potential mechanism of the miR-199a-5p induced inhibition on migration/invasion through EMT in breast cancer.

It has been well accepted that not all cells within tumor are identical and that in a number of different cancers, such as teratocarcinoma, a small sub-population of cancer cells possess “stem-like” characteristics including self-renewal, tumorigenicity, which defined as cancer stem cells (CSCs). In breast cancer, EMT has been associated with CSCs characteristics including self-renewal ability and the expression of stem cell-associated cell subpopulation CD44^+^/CD24^–/low^ [30]. Regarding the miR199-5p-induced EMT alteration in breast cancer, we further look into the stemness features related to miR-199a-5p. Single cell colony formation assay showed less and smaller colonies in miR-199a-5p group, suggesting its suppressive role in self-renewal. In recent years, tumor-initiating cells (TICs), have been widely implicated in different treatment-resistant tumors [31–33]. Recent evidence suggests that enhanced ALDH activity serves as a hallmark of CSCs measurable by the aldefluor assay. In breast cancer, the CSCs enriched in ALDH-positive cells isolated from human breast tumors were with high self-renewal and tumorigenic activity [34], indicating ALDH activity is an important and promising tool for the study of stem cells properties. We managed to discover that miR-199a-5p significantly reduced the CSCs/TICs population, which reflected by decline of the ALDH-positive cells. More recent findings identified that ALDH isoforms, particularly ALDH1A3, contributed to ALDH activity in breast CSC [35]. It has been widely reported that CD24-/CD44+ are the two cell surface markers used for isolating tumorigenic CSC from non-tumorigenic cancer cells [36, 37]. Our results demonstrated that miR-199a-5p indeed inhibited the breast cancer cell stemness by decreasing the CD24-/CD44+ population and ALDH activity.

Recent studies have suggested the possible relationship between miRNA deregulation and drug resistance. Preclinical models have proved that microRNA-based treatment work as an effective strategy in cancer treatment. MiR-451 and miR-27 have been implicated in the development of doxorubicin resistance [38, 39] while miR-326 was found to be associated with sensitivity to several chemotherapeutic drugs in breast cancer [40]. Recent studies have revealed that cisplatin treatment decreased miR-199a-5p expression in HCC patients, which may account for chemoresistance. Forced expression of miR-199a-5p promoted cisplatin-induced inhibition of cell proliferation, resulting sensitization of cancer cells to chemotherapy [41]. Also, miR-199a-5p is related to multidrug resistance in colon cancer [42] and it is upregulated in cisplatin-sensitive ovarian cancer cells [43]. In this study, we have established a robust tumor-suppressive role of miR-199a-5p in TNBC via modulation of EMT genes and stem cancer markers, further investigation effort should be placed on the altered chemotherapy sensitivity and hence might help in selecting a more suitable chemotherapeutic agent.

## Conclusions

Taken together, our results demonstrated that the TNBC-associated biomarker miR-199a-5p conferred tumor-suppressive role in breast cancer by regulating EMT process and stemness characteristics. Furthermore, ALDH1A3 may be a potential TNBC-specific candidate gene, which providing novel treatment options for breast cancer. However, further intensive investigation is needed to confirm its role in breast cancer.

## Abbreviations

ALDH: Aldehyde dehydrogenase
BAAA: Biodipy-aminoacetaldehyde
CSCs: Cancer stem cells
DAPI: 4′,6-diamidino-2-phenylindole
DCIS: Ductal carcinoma in situ
DEAB: Diethylaminobenzaldehyde
DMSO: Dimethyl sulfoxide
EMT: Epithelial-mesenchymal transition
ER: Estrogen receptor
HER2: Human epidermal growth factor receptor 2
IDC: Invasive ductal carcinoma
ILC: Invasive lobular carcinoma
MTT: (3-(4,5-dimethylthiazol- 2-yl)-2,5-diphenyltetrazolium bromide)
NA: Not applicable
PR: Progesterone receptor
qPCR: Quantitative polymerase chain reaction
TICs: Tumor-initiating cells
TNBC: Triple-negative breast cancer
UTR: Untranslated regions
